# A Comparative Study of Machine Learning and Deep Learning Models for Automatic Parkinson’s Disease Detection from Electroencephalogram Signals

**DOI:** 10.3390/diagnostics15060773

**Published:** 2025-03-19

**Authors:** Sankhadip Bera, Zong Woo Geem, Young-Im Cho, Pawan Kumar Singh

**Affiliations:** 1 Department of Information Technology, Jadavpur University, Jadavpur University Second Campus, Plot No. 8, Salt Lake Bypass, LB Block, Sector III, Salt Lake City, Kolkata 700106, India; sankhadipbera21@gmail.com (S.B.); pksingh.it@jadavpuruniversity.in (P.K.S.); 2 College of IT Convergence, Gachon University, Seongnam 13120, Republic of Korea; 3 Department of Computer Engineering, Gachon University, Seongnam 1342, Republic of Korea

**Keywords:** Parkinson’s disease detection, electroencephalogram signals, power spectral density, UC San Diego Resting State EEG dataset, IOWA dataset, support vector machine, convolutional neural network

## Abstract

**Background:** Parkinson’s disease (PD) is one of the most prevalent, widespread, and intricate neurodegenerative disorders. According to the experts, at least 1% of people over the age of 60 are affected worldwide. In the present time, the early detection of PD remains difficult due to the absence of a clear consensus on its brain characterization. Therefore, there is an urgent need for a more reliable and efficient technique for early detection of PD. Using the potential of electroencephalogram (EEG) signals, this study introduces an innovative method for the detection or classification of PD patients through machine learning, as well as a more accurate deep learning approach. **Methods:** We propose an innovative EEG-based PD detection approach by integrating advanced spectral feature engineering with machine learning and deep learning models. Using (a) the UC San Diego Resting State EEG dataset and (b) IOWA dataset, we extract a standardized EEG feature from five key frequency bands—alpha, beta, theta, gamma, delta (α,β,θ,γ,δ) and employ an SVM (Support Vector Machine) classifier as a baseline, achieving a notable accuracy. Furthermore, we implement a deep learning classifier (CNN) with a complex multi-dimensional feature set by combining power values from all frequency bands, which gives superior performance in distinguishing PD patients (both with medication and without medication states) from healthy patients. **Results:** With the five-fold cross-validation on these two datasets, our approaches successfully achieve promising results in a subject dependent scenario. The SVM classifier achieves competitive accuracies of 82% and 94% in the UC San Diego Resting State EEG dataset (using gamma band) and IOWA dataset, respectively in distinguishing PD patients from non-PD patients in subject. With the CNN classifier, our model is able to capture major cross-frequency dependencies of EEG; therefore, the classification accuracies reach beyond 96% and 99% with those two datasets, respectively. We also perform our experiments in a subject independent environment, where the SVM generates 68.09% accuracy. **Conclusions:** Our findings, coupled with advanced feature extraction and deep learning, have the potential to provide a non-invasive, efficient, and reliable approach for diagnosing PD, with further work aimed at enhancing feature sets, inclusion of a large number of subjects, and improving model generalizability across more diverse environments.

## 1. Introduction

Parkinson’s disease (PD) is a progressive, chronic, neurological disorder and the second most common malady that mainly affects the central nervous system of an individual, causing both motor and non-motor symptoms which significantly affect a person’s everyday life. The motor symptoms of PD include tremors, rigidity, bradykinesia, postural gait disturbances, etc. It also includes various non-motor symptoms like memory problems, mental health, and pain that impact patients’ quality of life. It was first described in 1817 by an English surgeon, James Parkinson, in his monograph “An Essay on the Shaking Palsy” [1]. Since then, the disease has been recognized as a complex condition with both motor and non-motor symptoms. Right now, after Alzheimer’s disease, it is the most common neurodegenerative disease. According to the current study, about 10 million people around the world (nearly 0.12% of the world population) are affected by PD. In the United States alone, nearly 90 thousand people are diagnosed with PD every year, which can rise up to 1.2 million by 2030 [2].

The motor symptoms of PD include tremors, rigidity, bradykinesia (slowness of movements), and postural instability. These symptoms often lead to difficulties with everyday tasks, impacting the quality of life. In addition, a variety of motor symptoms, like sleep disturbances, cognitive impairment, depression, anxiety, autonomic dysfunction, etc., are seen. Age is considered as the primary risk factor for PD. The prevalence of PD is nearly 1% in individuals over 65 years old, and it increases to nearly 4.3% in individuals over 85 years old. The exact causes of PD are still not properly known, but it is assumed that it arises from a combination of environmental and genetic influences.

Although Parkinson’s disease cannot be cured, early diagnosis is very crucial for managing symptoms and slowing disease progression, which improves the patient’s quality of life significantly [3]. Since no disease-modifying drugs have been approved to treat PD, this has become a major focus of PD research [4]. EEG (electroencephalography) signals offer a promising avenue for identifying neurological patterns. As EEG technology is reasonably cost-effective and portable, it is very useful to capture brain signal characteristics in real-world settings [5]. These signals are capable of capturing brain activity more quickly and over extended periods compared to other technologies [6]. The raw EEG data are complex and noisy, and need to be heavily pre-processed before feature extraction for the model training phase. Manifold machine learning and deep learning approaches have proven to be successful in analyzing these types of continuous signals [7]. However, studies specific to Parkinson’s disease detection have been limited.

In this study, we use two publicly available datasets, namely, (a) the UC San Diego Resting State EEG dataset and (b) the IOWA dataset, to observe the potential of EEG signals in detecting PD through both machine learning and deep learning approaches. By building traditional EEG processing methods like artifact removal, segmentation, feature extraction, etc., we extract key frequency band (α,β,θ,γ,δ) powers to train a robust model with both subject dependent and independent scenarios.

We initially use SVM (Support Vector Machine) as a baseline classifier by feeding the features matrix generated from each key frequency band individually to evaluate the initial effectiveness, which gives accuracy scores beyond 83% with the UC San Diego Resting State EEG dataset and more than 94% in the IOWA dataset (in the case of subject dependency), whereas an accuracy score of 68.09% is attained for a subject independent case.

**Figure 1:**
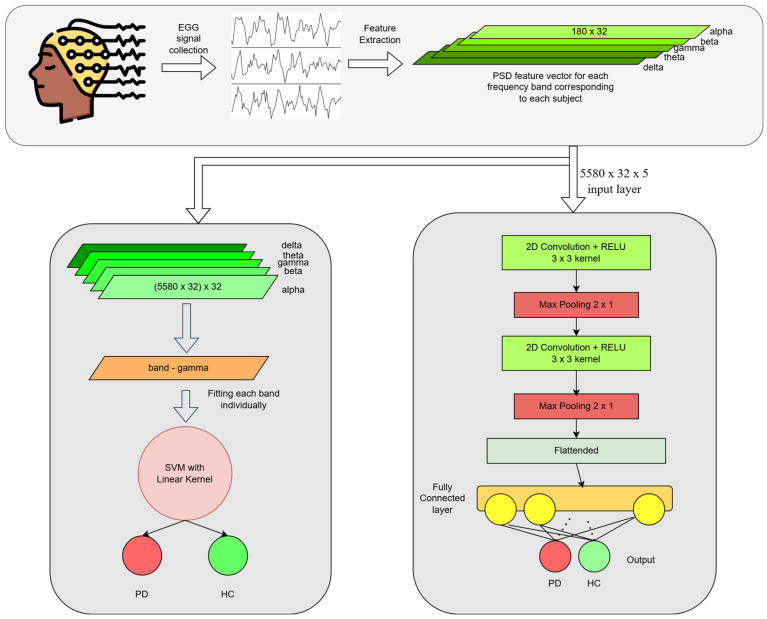
Diagram illustrating the complete pipeline of SVM (**left**) and our proposed CNN (**right**) model for PD detection from EEG signals.

However, to achieve more accurate and reliable results, we primarily focus on the convolution neural network (CNN), a deep learning model that effectively captures complex patterns inside the processed EEG data. With our proposed CNN model, we are able to achieve nearly 97% and 99% in those two datasets, respectively in a subject dependent case. These results showcase that our model performs very well across various time-series datasets. The brief flow of our work is showcased in Figure 1.

## 2. Related Study

Over the past few years, many studies in the medical field have increasingly focused on the application of computational methods for disease detection and monitoring or diagnosis. With the emergence of machine learning, deep learning, and other AI techniques, traditional diagnostic paradigms are being supplemented or replaced by data-driven approaches, which aim to offer a relatively more accurate detection of diseases.

In the context of neurological disorders, EEG is being used as a valuable tool for analyzing brain activity due to its high temporal resolution and non-invasive nature [8]. Prior studies have demonstrated the utility of EEG in detecting abnormalities associated with epilepsy and Alzheimer’s disease. Pirrone et al., 2022 [9] used the power spectrum, phase synchronization, and entropy measure of EEG signals for early detection of Alzheimer’s disease [10]. Emotion-based research has also benefited significantly from EEG. Several studies like Suhaimi et al., 2020 [11] revealed how EEG signals are utilized to classify emotional states, demonstrating the relationship between brainwave activity and affective processes. Dhara et al., 2023 [12] have described a fuzzy ensemble-based approach for emotion recognition based on EEG data with CNN, LSTM (Long Short-Term Memory), and GRU (Gated Recurrent Unit) models, which have achieved over 90% accuracy. Dave et al., 2024 [13] proposed an ensemble deep learning approach for improving the recognition accuracy of human emotions. Ghosh et al., 2022 [14] proposed an image-encoding technique for time-series data using deep neural network models for classification of the mental stress of a person. Mukherjee et al., 2024 [15] proposed a classification method of human emotions using EEG signals with a transfer learning approach.

PD-specific research has explored a variety of biomarkers, including motor assessments, voice analysis, and wearable sensors. Anjum et al., 2020 [16] proposed a novel approach utilizing a Linear Predictive Coding (LPC) approach to transform EEG signals into a set of distinctive features, achieving a classification accuracy of 85.7%, an AUC (area under the curve) of 85.2% on ROC (receiver operating characteristic) curves, and high levels of sensitivity and specificity, both at 85.7%. S.-B. Lee et al., 2022 [17] described a PD detection technique where they applied decision tree models with gradient boosting on the EEG data, achieving about 89.3% accuracy. Most recently, Lal et al., 2024 [18] introduced an innovative approach with various machine learning algorithms like KNNs (K nearest neighbors), XGBoost (Extreme Gradient Boosting), etc., by measuring the fractal dimension with several computational algorithms like Katz, Higuchi, etc., and achieved over 95% in some cases. Latifoğlu et al., 2024 [19] implemented SVM and other machine learning algorithms by applying the VKF (Vold-Kalman order filtering) method with the ten-fold leave-one-out cross-validation (LOOCV) process to EEG signals, achieving a perfect 100% accuracy in PD_OFF vs. HC classification.

Concerning deep learning techniques, Loh et al., 2021 [20] applied a two-dimensional CNN with Gabor transformation on the spectrograms generated from the EEG dataset in resting state to distinguish patients between PD_OFF and HC, achieving nearly 100% accuracy and 99.5% accuracy for patients with medication vs. healthy control classification. Qiu et al., 2022 [21] proposed a multi-scale CNN model with multi-pattern analysis for PD detection, which gives an accuracy score from nearly 98% to 99%. Qiu et al., 2024 [22] used a multi-scale CNN with prototype calibration to extract brain functional activities from EEG signals, achieving over 92% accuracy. Chang et al., 2023 [23] proposed an attention-based graph CNN extracting the log spectral power of different frequency bands for PD diagnosis using EEG, with an accuracy of 87.67%, while highlighting significant frontal lobe asymmetry in PD patients.

In EEG-based neurological disorder detection, several research gaps still remain. Studies such as Loh et al., 2021 [20] have demonstrated PD classification using CNN-based models which may be highly accurate, but it mostly relies on spectrogram representations instead of raw EEG features, which can cause the potential loss of critical time-domain information. In our work, we use raw EEG signals instead of the image encoding approach to prevent information distortion from the transformation process. Studies like Qiu et al., 2022 [21] have treated feature vectors from each of the frequency band individually, and analyzed them separately for classification tasks. This approach only captures distinct characteristics of individual frequency bands overlooking the interactions between them. In contrast, we stack the feature vectors across all five frequency bands, creating a more comprehensive input vector that incorporates both individual and combined spectral information, which allows the model to learn richer representations of EEG signals.

While most of the studies constructed a complex architecture for EEG-based PD detection, our approach demonstrates that a relatively simple model can achieve comparable performance.

## 3. Materials and Methods

### 3.1. Datasets

In this study, we utilize two public datasets:

- Dataset-1: “UC San Diego EEG Dataset”, recorded from patients suffering from PD in resting state from OpenNeuro.org [24];
- Dataset-2: “IOWA dataset”, from the “University of Iowa, Iowa, IA, USA” [16].

#### 3.1.1. Dataset-1

This dataset is acquired from the OpenNeuro platform and contains EEG signals from a total of 31 subjects; of which 15 are PD patients (7 male patients aged between 47 and 74 and 8 female patients aged between 52 and 71), and 16 are healthy controls or HCs (7 male patients aged between 57 and 82 and 9 female patients aged between 50 and 74). All the subjects belonging to this dataset are right-handed and have a mini-mental state score greater than 24, i.e., in normal cognition. All 15 PD patients have EEG records for both with medication (denoted as PD_ON) and without medication (denoted as PD_OFF). Therefore, a total of 46 (16 HCs and 2 EEG records for each of the 15 PD patients) EEG data are present in the dataset. An EEG system with 32 channels is used to record subjects’ data. The positions of these channels are as follows:

- Frontal and Prefrontal Regions: Fp1, Fp2, AF3, AF4, F3, F4, F7, F8, Fz;
- Frontocentral and Central Regions: FC1, FC2, FC5, FC6, C3, C4, Cz, T7, T8;
- Centroparietal and Parietal Regions: CP1, CP2, CP5, CP6, P3, P4, P7, P8, Pz;
- Parieto-Occipital and Occipital Regions: PO3, PO4, O1, O2, Oz.

The summary of this dataset is showcase in Table 1, further details can be found in [24].

#### 3.1.2. Dataset-2

This dataset was made by the University of Iowa, Narayanan Lab from 2017 to 2019. It contains EEG signals from a total of 28 subjects, of which 14 are PD (OFF, ON medication sessions are not specified) patients including 6 males and 8 females, aged between 54 and 86, and 14 are healthy controls (HCs) including 6 males and 8 females, aged between 54 and 86. This EEG dataset was collected with a 64-channel Brain-Vision system with a sampling frequency of 500 Hz. Out of these 64 channels, we selected 29 key bands in our experiments to maintain consistency between both datasets; these are Fp1, AF3, F7, F3, FC1, FC5, T7, C3, CP1, CP5, P7, P3, O1, Oz, O2, P4, P8, CP6, CP2, C4, T8, FC6, FC2, F4, F8, AF4, Fp2, Fz, Cz.

The summary of this dataset is showcase in Table 2, and further details can be found in [16].

### 3.2. Dataset Pre-Processing

EEG data that are picked up from the scalp contain a large amount of noise, which may soften some weaker EEG signals. Therefore, the raw EEG signals must be pre-processed. The entire pre-processing of raw EEG signals is carried out using the Python (version 3.9) MNE library [25]. We have extracted the first 180 s of EEG recordings from each subject from Dataset-1 and the first 120 s from Dataset-2. To prepare the EEG data for PD detection, a structured pre-processing pipeline is applied, retaining critical features and minimizing noise and irrelevant information for classification. The steps for EEG pre-processing are as follows:

#### 3.2.1. Band-Pass Filtering

Filtering is critical for maintaining the integrity of EEG data. First, the EEG signals from each channel undergo a band-pass filter, which filters the EEG signals between 0.5 and 50 Hz to isolate specific frequency bands that are known to carry information about cognitive and neurological states. We use band-pass filters to extract five key frequency bands:

1. Alpha (frequency range 8–13 Hz): Connected with calmness and wakeful rest;
2. Beta (frequency range 13–30 Hz): Related to problem-solving, active thinking, etc.;
3. Gamma (frequency range 30–48 Hz): Related to perception, attention, memory, consciousness, etc.;
4. Theta (frequency range 4–8 Hz): Linked to drowsiness, relaxation, etc.;
5. Delta (frequency range 1–4 Hz): Commonly associated with restorative processes and deep sleep [26].

#### 3.2.2. Artifact Removal

EEG signals often contain artifacts from eye blinks, muscle movements, etc. Artifact removal is a crucial part of EEG pre-processing. Here, we employ ICA (Independent Component Analysis) to isolate and remove artifacts, such as muscle movements, eye blinks, and other physiological noises from the EEG signals that can distort the data.

**Figure 2:**
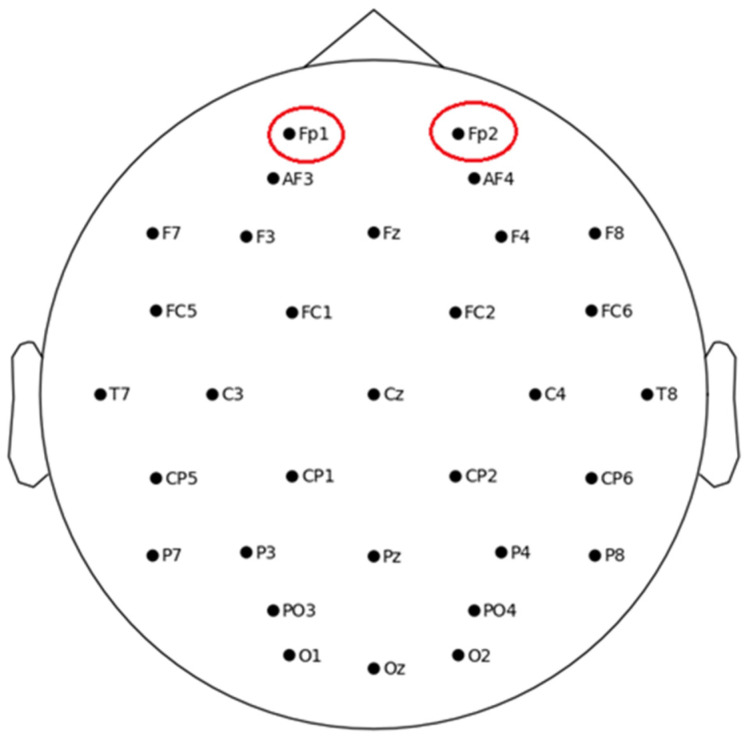
The physical location of sensors (2D) on the scalp with two EOG channels, “Fp1” and “Fp2”, marked with a red circle at the frontal lobe.

To detect eye blink artifacts in EEG data, we used some electrooculography (EOG) channels or EEG channels, which are relatively more sensitive to eye movements. In this case, “Fp1” and “Fp2” are the two frontopolar EEG channels located on the forehead near the eyes (refer to Figure 2) and are highly sensitive to eye blinks and movements. These are ideal for detecting artifacts caused by horizontal blinks or eye movements.

#### 3.2.3. Segmentation

As different EEG signals from different subjects have dissimilar lengths, segmentation is necessary to standardize the length data. Following ICA, the EEG signals are divided into 180 segments for Dataset-1 and 120 segments for Dataset-2 with a time sample of 1 s. Now, these uniformly trimmed data for each subject have a shape of 180 × 32 × 512 (time samples × #channels × sampling points) for Dataset-1, and 120 × 32 × 500 (time samples × #channels × sampling points) for Dataset-2.

#### 3.2.4. Extraction of Features

In the context of PD, PSD (power spectral density) is a powerful tool for analyzing the frequency characteristics of various signals such as tremors, EEG, voice, etc. It is very effective in identifying shifts in brain activity associated with PD, especially with EEG [25]. PSD captures the power densities across all frequency bands of EEG signals [16]. Abnormalities in these bands, such as increased beta activity or reduced alpha activity or increased theta power and reduced beta power, are associated with PD and can aid in diagnosis and monitoring disease progression. Also, the PSD of EEG analysis is a low-cost, non-invasive method for PD detection. Several studies have identified altered EEG spectral patterns, making the measurement of PSD a valuable feature for disease classification.

PSD is measured using Welch’s method, which reduces noise by averaging over multiple segments of the signal.

The PSD S(f) of a signal x(t) is mathematically defined as follows:

(1) $$Sf\;\;\underset{T\to \infty }{\lim}\frac{1}{2T}{\left|\int_{-T}^{T}x\left(t\right)e^{-j2\pi ft}dt\right|}^{2},$$

where f represents the frequency and x(t) is the EEG signal.

**Figure 3:**
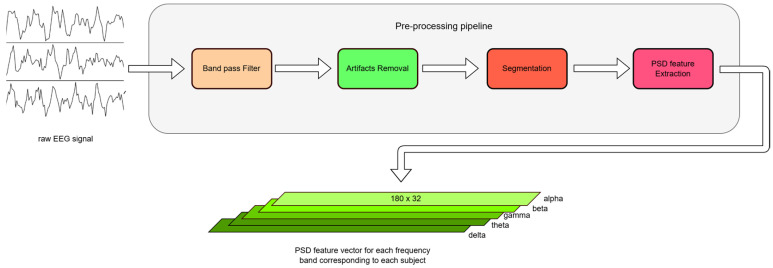
Pre-processing pipeline for raw EEG signals used in our proposed work.

Here, from each 1 s segment, the PSD features are computed across each frequency band (α,β,θ,γ,δ), generating a feature vector where each channel has a set of PSD values for each frequency band, transforming the data shape (180 × 32 for Dataset-1 and 120 × 32 for Dataset-2) to a feature matrix for model input. The whole pre-processing steps are illustrated in Figure 3.

### 3.3. Classification

In this section, we discuss two techniques based on the PSD feature vectors generated from the pre-processed EEG signals. We implement (a) a machine learning model, which includes the traditional SVM classifier and (b) a deep learning model involving CNN with a relatively complex input format. We perform the classification test for the following three cases:

- Case 1: PD_ON vs. HC (distinguishing PD patients with medication from non-PD patients);
- Case 2: PD_OFF vs. HC (distinguishing PD patients without medication from non-PD patients);
- Case 3: PD vs. HC (distinguishing PD patients from non-PD patients).

Here, case 1 and case 2 are conducted on Dataset-1 and Case 3 is conducted on Dataset-2.

#### 3.3.1. Machine Learning Baseline

Here, we implement the popular SVM or Support Vector Machine with the linear kernel as a baseline classifier, which gives the binary classification between PD and non-PD patients for all three cases (case 1, case 2, and case 3). In our experiment, all the HCs are labeled as “0”, and PDs are labeled as “1”. We then classify them using different PSD features extracted from each of the bands (as there are five frequency bands, there are five separate input feature vectors, each having a similar shape). Features are extracted for each of the 32 EEG channels [21], capturing signal power across different brainwave frequencies.

The PSD feature matrix for each subject in each band has a size of 180 × 32 (time samples × number of channels); therefore, the shape of the feature matrix for a classification with Dataset-1 becomes 5580 × 32 ([180 × 15 (PD_ON/OFF) + 180 × 16 (HC)] × 32 channels). We split the whole dataset into a 9:1 ratio for training and testing purposes. Therefore, the training segment becomes 5022 × 32 in size for each band, and the test segment becomes 558 × 32 for each band.

For a subject dependent case, instead of merging the time frames, we maintained the shape of the dataset, where data for each subject in each band have a size of 180 × 32 (time samples × number of channels) for Dataset-1 and 120 × 29 (time samples × number of channels) for Dataset-2. Then, we perform a three-fold cross-validation with a random state of 42.

We apply StandardScaler to normalize the feature vectors into a standard Gaussian distribution with the “0” mean and “1” unit of standard deviation.

**Figure 4:**
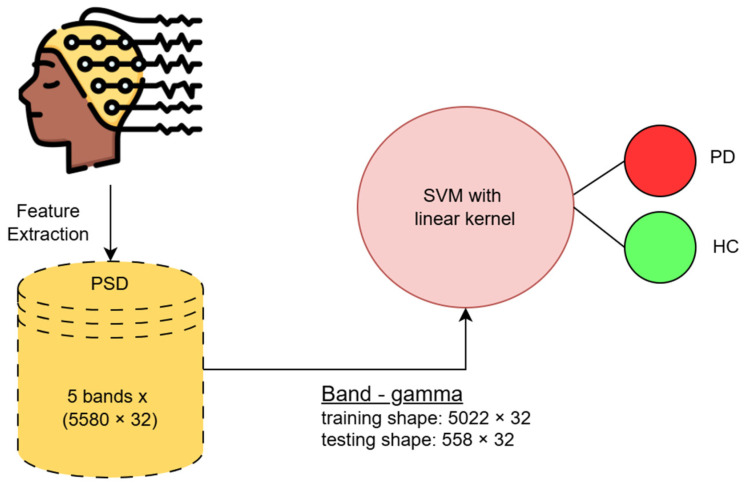
Pictorial representation of our machine learning framework based on the SVM classification model utilizing the Gamma band.

The architecture of SVM classifier is illustrated in Figure 4.

#### 3.3.2. Deep Learning Classification

**Figure 5:**
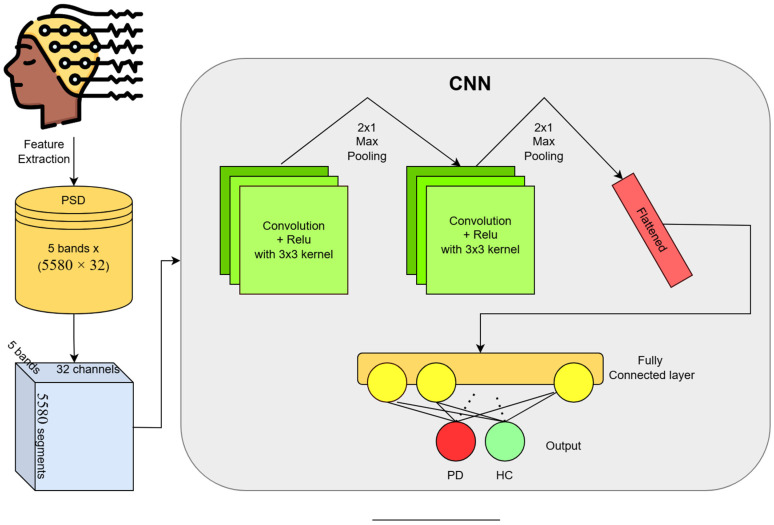
Our proposed two-dimensional CNN architecture with two 3 × 3 convolutional layers and two 2 × 1 max pooling layers following one FC layer.

To detect the PD more accurately, we implement a deep CNN model to capture complex patterns inside EEG signals. In the field of deep learning and healthcare, CNN has performed very well, not only with image-type data, but also with various time-series data related to the human body [27,28,29]. In some recent studies [30], CNN models have shown effectiveness in automatic PD detection using raw EEG signals. Figure 5 shows the arrangements of our deep convolutional model. We combine the features from all five bands to create a 2D (#bands × #channels) input vector for each time sample, allowing the CNN to learn from all frequency bands at once. The expanded shape of the feature map becomes (5580, 32 × 5).

For the subject dependent case, we stack data for each frequency band, where data for each subject have a size of 180 × 160 (time samples × (number of channels × #bands)) for Dataset-1 and 120 × 145 (time samples × (number of channels × #bands)) for Dataset-2. Then, we perform a five-fold cross-validation with a random state of 42.

We apply StandardScaler to normalize the feature vectors into a standard Gaussian distribution with the “0” mean and “1” unit of standard deviation.

The architecture is the same for both cases, and can be summarized as follows:

1. Input Layer: Our model inputs a feature matrix of shape 5580, 32 × 5 (number of segments, channels × bands), where each segment represents a 1 s epoch of EEG data with PSD values from 32 channels and five bands.
2. Convolutional Layers: Two convolutional layers with 3 × 3 kernels are stacked to extract spatial features across channels and bands. Then, Rectified Linear Unit (ReLU) activations are applied to these layers to introduce some non-linearity.
3. Pooling Layers: We used two max pooling layers of 2 × 1 in size, resulting in an output shape of 16 × 5 in each segment.
4. Fully Connected Layers: After two pairs of convolutional and max pooling layers, the flattened feature map is fed into a fully connected layer (FC) with the same activation ReLU. These layers integrate the high-level features learned from the convolutions to produce a final classification decision.
5. Output Layer: A sigmoid layer is used as an output layer for binary classification, which generates the probability of each class (PD or healthy control).

We train the model using the Adam optimizer, which is relatively efficient and faster and the binary cross-entropy loss for loss function, with a five-fold cross-validation technique for better generalizability.

## 4. Experimental Results

This section outlines the findings from our experiments, comparing the baseline machine learning approach (using SVM) and the deep learning model on EEG data-based PD detection. We performed all the experiments using an ASUS TUF F15 laptop with 16 GB DDR4 Random Access Memory (RAM) and a dedicated RTX 3050 graphics card of 4 GB memory with an 11th Gen Intel 11400 h processor. Windows 11 OS is used as an experimental platform. The entire coding for this experiment is written in Microsoft Visual Studio code and Jupyter Notebook (v7.3.3).

We first perform binary classification for case 1 and case 2 on Dataset-1 and case 3 classification on Dataset-2. All the experiments are conducted using the PSD features extracted from α,β,θ,γ,δ frequency bands. We use several performance metrics, like accuracy, specificity, sensitivity, etc., to evaluate our model. The details about these metrics can be found at (Goutte and Gaussier, 2005 [31]).

### 4.1. Results Using Machine Learning-Based Classification

We use a traditional SVM as a baseline classifier based on the PSD feature vector from individual frequency bands. Each feature vector (shape 5580 × 32) is treated independently to classify PD patients and HCs. Table 3 shows the two-class classification result based on PSD features for five frequency bands using SVM with subject dependency for all cases.

While the SVM classifier provided a reasonable starting point, its ability to capture complex EEG patterns is limited for some bands, but it can be seen that some bands like gamma (which is related to perception, attention, memory, consciousness, etc.) and beta (which is related to active thinking) are effectively classifying between PDs and HCs in both datasets. For the classification of beta and gamma bands, the accuracy is more than 80% in Dataset-1 (case 1 and case 2), and it reaches above 90% in Dataset-2 (case 3).

While the subject dependency may cause some biases, we also train our SVM model on EEG data from individual subjects to assess its ability to detect PD when trained on a single person’s data. The model achieved average classification accuracies of 68.09% and 64.54% with theta and alpha bands, respectively, while the performance of other bands is significantly less. This result is very much deviating from the result we achieved with a subject dependent scenario, where gamma and beta bands are performing well as compared to others. The detailed performance for each band is summarized in Table 4.

### 4.2. Results Using Deep Learning-Based Classification

For more accurate results, we implement a deep two-dimensional CNN-based model to classify PD patients and HCs for the same three cases based on a modified PSD feature matrix. The model incorporates a sigmoid activation function in the output layer and is evaluated using the five-fold cross-validation for robust performance measurement. Table 5 shows the classification results considering subject dependency for all three cases.

From Table 5, we can observe that our proposed CNN model does an excellent job over the traditional SVM model in predicting PDs and HCs. The combined features from all the five bands help the model to achieve an accuracy up to 96–97% in Dataset-1, while it reaches up to 99% in Dataset-2.

**Figure 6:**
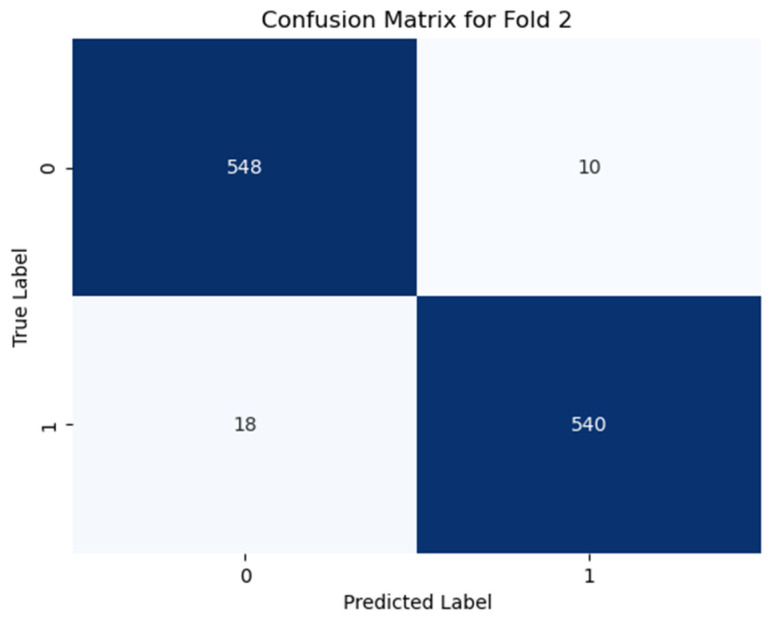
Confusion matrix for case 1 in fold 2, where “0” denotes non-PD and “1” denotes PD. The model correctly predicts 97.5% of the samples (subject dependent).

**Figure 7:**
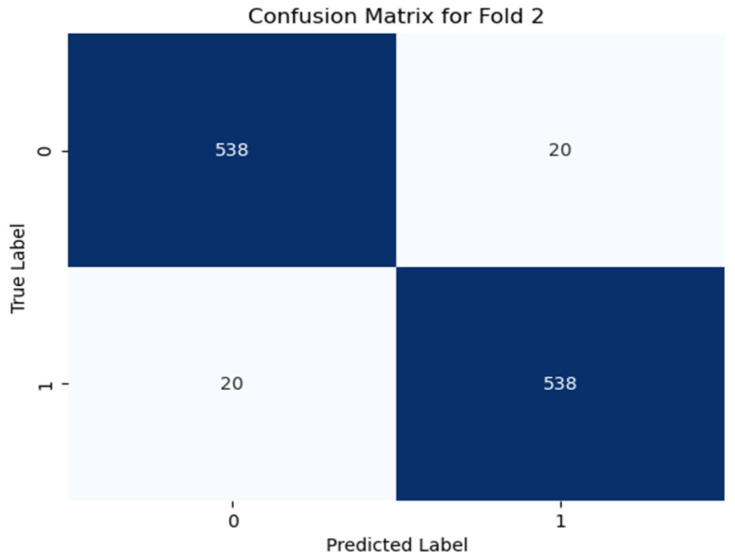
Confusion matrix for case 2 in fold 2, where “0” denotes non-PD and “1” denotes PD. The model correctly predicts 97% of the samples (subject dependent).

**Figure 8:**
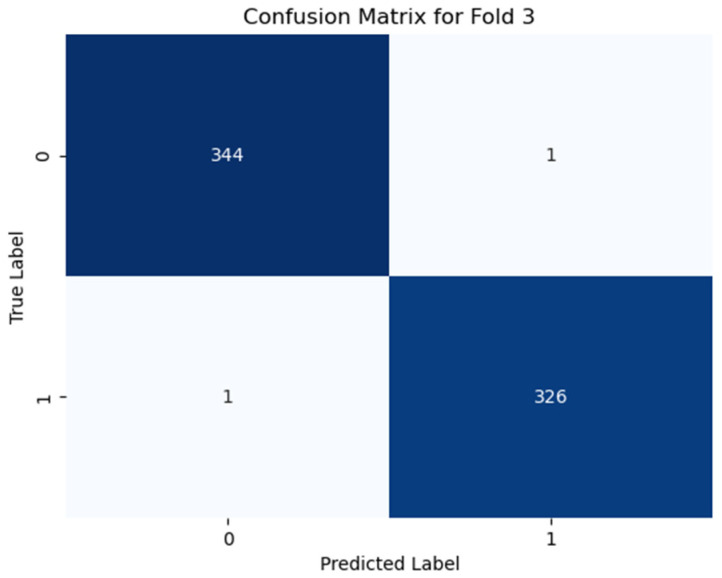
Confusion matrix for case 3 in fold 3, where “0” denotes non-PD and “1” denotes PD. The model is giving a wrong prediction for two samples only (subject dependent).

Additionally, the confusion matrices for all the folds are analyzed to identify common misclassification patterns, which are shown (only best the fold) in Figure 6, Figure 7 and Figure 8. The confusion matrix for using Dataset-1 for case 1 (best fold) is shown in Figure 6, where the model correctly predicts 548 samples as negative, which are actually negative (fold 2, Figure 6), and 540 samples as positive, which are truly positive, while it only gave some incorrect predictions like where the class is actually negative, but the model predicts as positive giving a 97.5% accuracy score for this fold. In case 2, the model correctly classifies nearly 97% of samples correctly (538 true negatives, 538 true positives in fold 2 as shown in Figure 7), giving an average accuracy of about 95.81% for all the folds. For case 3 classification in Dataset-2 (illustrated in Figure 8), the model performs very well, where it detects more than 99% of samples correctly.

**Figure 9:**
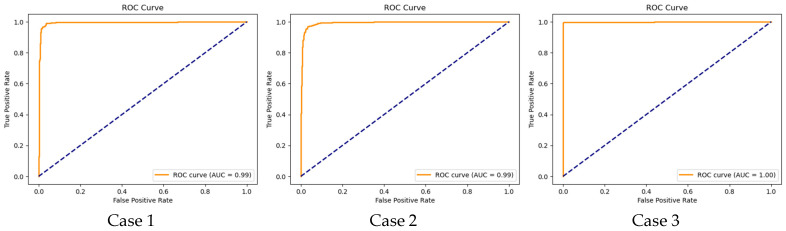
ROC curves for UC San Diego Resting State EEG and IOWA datasets showing model performance across all threshold values in all three cases (subject dependent).

**Figure 10:**
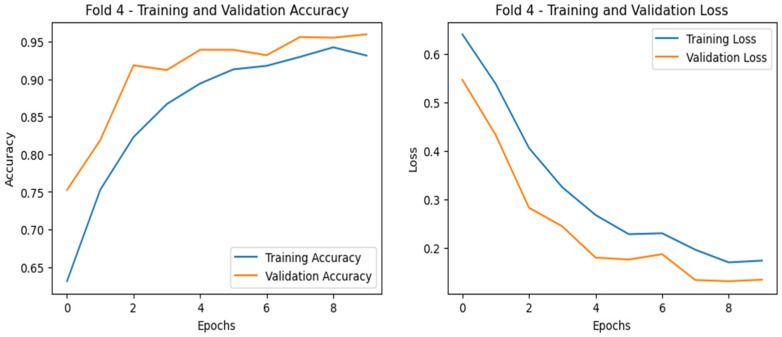
Training and validation curves for case 1 starting with a relatively large gap, and the gap seems to be decreasing as the number of epochs increases, indicating that the model is not significantly overfitting, i.e., better generalization (subject dependent).

**Figure 11:**
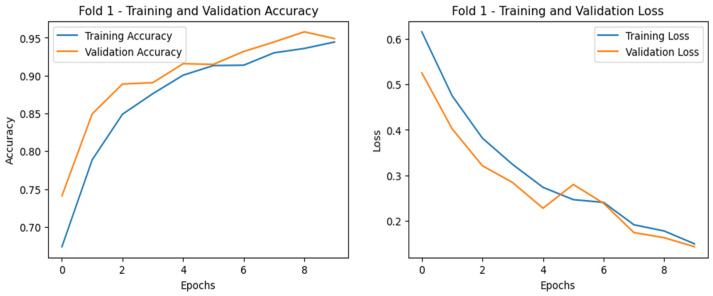
Training and validation curves for case 2 starting with a smaller gap, but the gap decreases as the number of epochs increases, and at some point, those two curves intersect, indicating a potential turning point in generalization (subject dependent).

**Figure 12:**
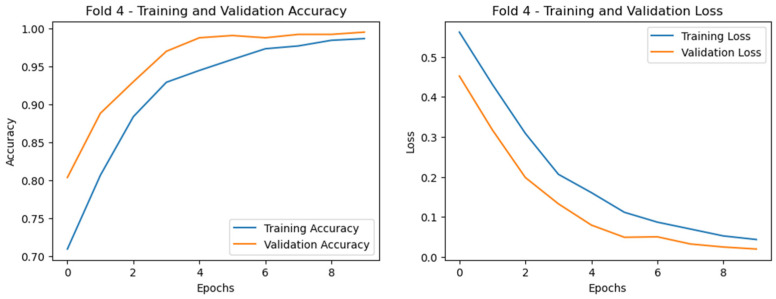
Training and validation curves for case 3 showing the model convergence after eight epochs with minimal overfitting since the gap between the two curves is considerably decreasing (subject dependent).

To further illustrate the performance, we plotted the ROC curves (illustrated in Figure 9) as well as training and validation curves for the best fold (highlighted in Figure 10, Figure 11 and Figure 12), showing the significant improvement of sensitivity and specificity with the proposed deep CNN model.

The AUC is nearly 0.99 for both case 1 and case 2, indicating that our proposed CNN model is effectively leveraging the complex patterns from EEG signals to accurately identify PD patients from non-PD patients, suppressing the performance of the conventional SVM model. For Dataset-2 (case 3), the model demonstrates exceptional performance, perfectly discriminating the PD patients from non-PD patients. The high AUC value (AUC “1”) confirms the robustness and accuracy of the model.

The training and validation curves for all three cases are shown in Figure 10, Figure 11 and Figure 12, respectively, where the accuracy curves are shown in the left part and the loss curves are shown in the right part for the best fold. For case 1 classification inn Dataset-1 (Figure 10), the training accuracy seems to start lower at around 63–65% and increases steadily, reaching ~93–95%, and the validation accuracy starts higher at around 78–80% and increases steadily, stabilizing at ~97%. The steady rise and minimal gap between training and validation accuracy curves imply that no significant overfitting is occurring. The loss curves decrease consistently, with the validation loss being slightly lower, suggesting that the model is not overfitting and is performing well.

For case 2 (Figure 11), the gap between training and validation curves is smaller at the initial point, and gradually, the curves become similar in some cases, indicating that the model is generalizing well with no overfitting or underfitting. For case 3 (PD vs. HC) classification in Dataset-2 (Figure 12), the training and validation accuracy curves achieved normalization at nearly 99%, and for the loss, both curves converged at below 5%, indicating the excellent performance of the model.

For further exploration, we again perform the same experiment in a subject independent case. However, due to the limited sample size, the CNN struggles to generalize effectively. The CNN usually requires large datasets to capture robust spatial and temporal patterns in EEG signals. Though it generates an average accuracy from 61% to 64%, it fails to classify a particular class every time. The lack of sufficient training samples per subject hindered the model’s ability to extract stable features from the dataset.

### 4.3. Ablation Study: Impact of Model Components

We conducted an ablation study to assess the effectiveness of our proposed model, comparing its performance against a 1D-CNN model for a subject dependent scenario only, in which we trained separately on each frequency band. This evaluation helps to determine whether learning from a combined feature matrix provides a performance advantage over using them individually.

#### Experimental Setup

- The 2D-CNN Model: Trained using the combined features from all five frequency bands;
- The 1D-CNN Model: Five separate models trained individually on five separate bands.

The classification accuracy for each experiment is shown in Table 6.

It can be seen from Table 6 that our 2D-CNN model outperforms all other one-dimensional models, achieving an overall higher classification accuracy (96.70% for case 1, 95.81% for case 2, 99.29% for case 3) by leveraging information from all EEG bands simultaneously. Among the 1D-CNN models, the gamma band provides the highest accuracy of 98.31% (in case 1), suggesting its significance in PD classification.

The results demonstrate how the combining PSD features from multiple frequency bands help to discover inter-band dependencies, resulting in a great classification performance.

## 5. Discussion

In summary, this study reviewed the effectiveness of EEG-based machine learning (SVM), as well as more effective deep learning (CNN) approaches for non-invasive PD detection for both subject dependent and independent scenarios. Our study mostly focuses on PSD features from five different frequency bands, as the PSD represents the distribution of signal power across different regions of the brain [32].

### 5.1. Significance of the Findings

Our analysis on PSD features reveals that different frequency bands contribute variably in PD classification. From the SVM classification result, we can observe that bands like gamma and beta yield higher accuracy, while bands like delta, theta, and alpha are not classifying PD patients from non-PDs. This result indicates that relatively higher frequency bands play a more crucial role in distinguishing PD patients than lower frequency bands. These findings help in selecting the optimal frequency bands for EEG-based PD detection (in our case gamma and beta bands). Since SVM is treating the feature matrix from each band individually, it may fail to capture some important cross-frequency dependencies.

However, our SVM model outperforms some of the existing work (e.g., Qiu et al., 2022 [21]) involving SVM for PD detection by giving better classification results with over 80% accuracy in Dataset-1 and over 94% accuracy in Dataset-2 (see Table 3). The abovementioned finding is implying that our method of pre-processing (such as the Independent Component Analysis (ICA) for artifact removal, band-pass filtering, and custom segmentation, etc.) ensured high-quality input data, contributing significantly to model performance.

### 5.2. Strengths of the Deep Learning Approach

In the context of the medical field, our results need to be highly accurate; therefore, we primarily focused on the deep learning model over the regular techniques of machine learning. Here, instead of treating features related to each band individually, we stacked features from all the frequency bands (α,β,θ,γ,δ) together to build a rich feature vector. This approach incorporates the combined spectral information instead of distinct characteristics of different frequency bands. With this integration, our model is able to learn richer representations from EEG signals, improving classification performance and robustness. With this structured dataset, the model can leverage inter-frequency dependencies, which are often crucial for distinguishing complex patterns from brain activity.

In both datasets, the model showed high classification performance (see Table 5). Moreover, the model’s performance across the five-fold cross-validation indicates its robustness to variations in the training data, which is essential for deployment in real-world scenarios. In Dataset-1 (case 1), the model gives an accuracy score of 96.70%, precision of 95.9%, sensitivity of 97.38%, F1-score of 96.62, AUC of 99.29%, and with Dataset-2, the classification accuracy is 99.29%, precision is 99.24%, sensitivity is 99.36%, F1-score is 99.29, and AUC is 99.91%.

The improvement in performance highlights the effectiveness of the deep learning model, like CNN, in leveraging the complex patterns and cross-frequency dependencies contained within raw EEG signals. The inclusion of several 3 × 3 convolutional layers allowed the model to extract meaningful patterns across EEG channels and frequency bands, which traditional machine learning models, like SVM, cannot fully utilize.

This performance result is not only superior to the SVM, but also matches the accuracy over other previous studies which involve a relatively more complex CNN. The performance comparison of existing PD detection (related to the subject dependent scenario) methods with our proposed methods is shown in Table 7 and Table 8.

### 5.3. Limitations

Despite its strong performance, our model has some limitations. The number of subjects in the standard datasets is quite small (only 15 PD patients, 16 HCs in Dataset-1, and only 14 PD patients, 14 HCs in Dataset-2). A large dataset (many PD patients) is necessary in better model generalizability across different stages of PD. This limitation is very much reflected when we performed our experiments with subject independency, where the deep learning model nearly fails to give any significant effect. Despite this small sample size, the training process requires a relatively higher amount of computational resources, which may not always be practical in environments with limited resources. Second, the model focuses only on PSD features; while effective, it may overlook other potentially informative non-linear features, such as entropy, phase-locked value (PLV), or wavelet-based measures, and we have not utilized any statistical measures in our work. Future work should explore the integration of these additional features to further enhance performance.

### 5.4. Future Directions

To achieve more promising results, our future research should focus on several key areas:

- Use of data augmentation techniques, transfer learning approaches, and inclusion of more diverse populations into the dataset to improve model generalizability.
- Incorporating advanced feature extraction techniques, such as wavelet transforms and entropy measures, along with other statistical measures like standard deviation, Kurtosis, etc., to capture more nuanced signal characteristics.
- Exploring alternative models, such as LSTM, to capture more complex sequences of EEG signals and attention mechanisms to better account for temporal dependencies in EEG signals.
- Employing transfer learning from pre-trained models, or hybrid architectures combining CNNs with traditional feature-based methods with subject independency to improve performance on small EEG datasets.

With these further refinements, our model could serve as a practical tool for early diagnosis, disease monitoring, disease detection, etc., of Parkinson’s disease in clinical settings.

## 6. Conclusions

In this study, we investigate the application of both SVM and deep CNN approaches for detecting Parkinson’s disease based on the functional activity of PD patients related to the brain, offering a non-invasive and efficient diagnostic approach. In the medical domain, accurate predictions are very essential. For instance, these technologies could be used to monitor and assess the emotional states of patients with mental health conditions, providing valuable insights about personalized treatment plans [12]. With the conventional SVM technique, we are able to achieve an average accuracy score of 80%, which goes up to 94% in some cases. Though this study is matching the current state of the art, the performance is not sufficient. Therefore, we implemented a deep, optimized two-dimensional CNN model with the modified feature matrix. We tested our proposed deep CNN model with a five-fold cross-validation, which has performed significantly well, demonstrating superior accuracy, sensitivity, specificity, etc. (all ranges from 96% to 99%). By leveraging the PSD (power spectral density) features from five different frequency bands and with robust pre-processing techniques, the model effectively captured neurophysiological patterns indicative of Parkinson’s disease (including both with medication and without medication). The majority of the studies used complex architectures, which not only take more time to execute, but also are resource-consuming, whereas with this relatively less complex architecture, we are able to match the current state of the art even better in some cases.

However, challenges like the limited number of samples in the datasets, other feature extraction techniques, more hyperparameter tuning, etc., must be addressed to enhance real-world applicability. Future research focusing on these issues could further improve model generalization and diagnostic utility.

In conclusion, this research advances in leveraging EEG-based deep learning techniques with a baseline SVM classifier for early detection of PD. Our findings highlight the importance of high-frequency bands like gamma and beta in distinguishing PD patients from non-PD, which could aid in refining diagnostic criteria. These insights could contribute to the development of automated, EEG-based diagnostic tools which is not only high performing, but also cost effective and scalable. Our approach has the potential to improve disease management and can enable early intervention in real-world healthcare settings providing a simpler and reliable EEG-based diagnostic tool for PD.

## Figures and Tables

**Table 1:** Subject demographics of Dataset-1.

	PD	HC
Number of subjects	15	16
Gender	7 males–8 females	7 males–9 females
Age range	47 to 74	57 to 82
Mini-mental state score (avg.)	28.9	29.2

**Table 2:** Subject demographics of Dataset-2.

	PD	HC
Number of subjects	14	14
Gender	6 males–8 females	6 males–8 females
Age range	54 to 86	54 to 86
Montreal cognitive assessment (avg.)	25.9	27.2

**Table 3:** The classification result using SVM, showing accuracy, specificity, and sensitivity values for all cases on their respective datasets (subject dependent).

	Case 1 (Dataset-1)			Case 2 (Dataset-1)			Case 3 (Dataset-2)		
	Accuracy	Specificity	Sensitivity	Accuracy	Specificity	Sensitivity	Accuracy	Specificity	Sensitivity
theta	0.7744	0.8438	0.7004	0.7819	0.8691	0.6889	0.8176	0.8827	0.7524
alpha	0.7900	0.8781	0.6959	0.8002	0.8854	0.7093	0.8429	0.8810	0.8048
beta	0.8206	0.8503	0.7889	0.8116	0.8330	0.7889	0.9042	0.9238	0.8845
gamma	0.8228	0.7872	0.8607	0.8066	0.8792	0.7293	0.9414	0.9589	0.9238
delta	0.6559	0.6795	0.6307	0.6975	0.8420	0.5433	0.6815	0.8458	0.5173

**Table 4:** The classification result using SVM, showing accuracy, specificity, and sensitivity values for all cases on their respective datasets (subject independent).

	Case 1 (Dataset-1)	Case 2 (Dataset-2)	Case 3 (Dataset-3)
Accuracy			
theta	68.09%	48.48%	52.96%
alpha	61.42%	64.54%	56.66%
beta	45.71%	45.15%	49.63%
gamma	48.18%	51.51%	46.29%
delta	55.23%	48.48%	49.62%

**Table 5:** Results of a two-class classification using our proposed deep CNN model showing accuracy, precision, sensitivity, F1-score, and AUC scores for all cases on their respective datasets (subject dependent).

	Dataset-1		Dataset-2
	Case 1	Case 2	Case 3
Accuracy	0.9670	0.9581	0.9929
Precision	0.9590	0.9511	0.9924
Sensitivity	0.9738	0.9633	0.9936
F1-Score	0.9662	0.9570	0.9929
AUC	0.9929	0.9919	0.9991

**Table 6:** Ablation study of our proposed model against a 1D-CNN model considering different frequency bands (subject dependent). The best result is shown in bold text.

Model	EEG Band	Case	Accuracy
1D-CNN	theta	Case 1 Case 2 Case 3	71.78% 69.41% 90.74
1D-CNN	alpha	Case 1 Case 2 Case 3	64.95% 64.28% 90.05%
1D-CNN	beta	Case 1 Case 2 Case 3	79.37% 75.77% 92.39
1D-CNN	gamma	Case 1 Case 2 Case 3	**98.31%** 85.34% 97.22%
1D-CNN	delta	Case 1 Case 2 Case 3	70.51% 58.50% 85.05
2D-CNN	All five bands combined	Case 1 Case 2 Case 3	96.70% **95.81%** **99.29%**

**Table 7:** Comparison of existing machine learning PD detection methods with our proposed methods (all performance measures are taken from the respective authors’ experiments).

Work	Dataset	Model	Classification	Accuracy
Lal et al., 2024 [18]	Dataset-1	KNN classifier with the Higuchi fractal dimension	Case 1 Case 2	96.46% 94.45%
Latifoğlu et al., 2024 [19]	Dataset-1	SVM with LOOCV	Case 1 Case 2	100% 99.68%
		KNN with LOOCV	Case 1 Case 2	99.68% 100%
Qiu et al., 2022 [21]	Dataset-1	SVM based on power spectral density features	Case 1 Case 2 Case 3	82.33% 78.69% 78.08%
	Dataset-2			
S.-B. Lee et al., 2022 [17]	Dataset-2	Decision Tree with gradient boost using the Hjorth parameter	Case 3	89.30%
Our proposed method	Dataset-1	SVM based on power spectral density features	Case 1 Case 2 Case 3	82.28% 81.16% 94.14%
	Dataset-2			

**Table 8:** Comparison of existing deep learning PD detection methods with our proposed methods (all performance measures are taken from the respective authors’ experiments).

Work	Dataset	Model	Classification	Accuracy
Qiu et al., 2024 [22]	Dataset-1	Multi-scale CNN with prototype calibration	Case 1 Case 2 Case 3	88.7% 84.5% 83.2%
	Dataset-2			
Chang et al., 2023 [23]	PD Oddball Data	Attention-Based Graph CNN with the LOOCV method	Case 1 Case 2	79.96% 87.67%
Qiu et al., 2022 [21]	Dataset-1	CNN (multi-layer perceptron with weight sharing)	Case 1 Case 2 Case 3	98.97% 97.15% 99.82%
	Dataset-2			
Loh et al., 2021 [20]	Dataset-1	CNN with Gabor transformation of EEG	Case 1 Case 2	100% 99.44%
Our proposed method	Dataset-1	2D-CNN	Case 1 Case 2 Case 3	96.70% 95.81% 99.29%
	Dataset-2			

## Data Availability

The two most popular datasets are utilized in this study; these datasets are available on the internet. 1. The UC San Diego dataset from OpenNeuro. Link: https://openneuro.org/datasets/ds002778/versions/1.0.4 (accessed on 1 December 2024). 2. Iowa dataset from the University of Iowa, Narayanan Lab. Link: https://www.dropbox.com/scl/fi/a5ib07b51iutjrr20dryx/public-folder-Fahim-Anjum.zip?rlkey=ulq1416i7l9fi0ckxooakzzh1&e=1&dl=0. The source codes related to this work can be found by accessing the following GitHub (version 2.25.2) link: https://github.com/Sankhadip-007/Detecttion-of-Parkinson-s-Disease-using-machine-learning-and-deep-learning.

## Abbreviations

The following abbreviations are used in this manuscript:

PD	Parkinson’s disease
SVM	Support vector machine
CNN	Convolutional neural network
PSD	Power spectral density
EEG	Electroencephalogram
PLV	phase-locked value

## References

[1] Goetz C.G. (2011). The History of Parkinson’s Disease: Early clinical descriptions and neurological therapies. Cold Spring Harb. Perspect. Med..

[2] Statistics. Parkinson’s Foundation. https://www.parkinson.org/understanding-parkinsons/statistics.

[3] Ugrumov M. (2020). Development of early diagnosis of Parkinson’s disease: Illusion or reality?. CNS Neurosci. Ther..

[4] Mari Z., Mestre T.A. (2022). The Disease Modification Conundrum in Parkinson’s Disease: Failures and Hopes. Front. Aging Neurosci..

[5] Biasiucci A., Franceschiello B., Murray M.M. (2019). Electroencephalography. Curr. Biol..

[6] Aljalal M., Aldosari S.A., AlSharabi K., Abdurraqeeb A.M., Alturki F.A. (2022). Parkinson’s Disease Detection from Resting-State EEG Signals Using Common Spatial Pattern, Entropy, and Machine Learning Techniques. Diagnostics.

[7] Mei J., Desrosiers C., Frasnelli J. (2021). Machine learning for the diagnosis of Parkinson’s disease: A review of literature. Front. Aging Neurosci..

[8] Hari R., Puce A. (2017). MEG-EEG Primer.

[9] Pirrone D., Weitschek E., Di Paolo P., De Salvo S., De Cola M.C. (2022). EEG signal processing and supervised machine learning to early diagnose alzheimer’s disease. Appl. Sci..

[10] Palop J.J. (2009). Epilepsy and cognitive impairments in Alzheimer disease. Arch. Neurol..

[11] Suhaimi N.S., Mountstephens J., Teo J. (2020). EEG-Based Emotion Recognition: A State-of-the-Art review of current trends and opportunities. Comput. Intell. Neurosci..

[12] Dhara T., Singh P.K., Mahmud M. (2024). A fuzzy ensemble-based deep learning model for EEG-based emotion recognition. Cogn. Comput..

[13] Dave M., Mukherjee S.K., Singh P.K., Mahmud M., Mahmud M., Kaiser M.S., Bandyopadhyay A., Ray K., Al Mamun S. (2024). Deep Ensemble Learning Approach for Multimodal Emotion Recognition. Proceedings of the 3rd International Conference on Trends in Electronics and Health Informatics (TEHI-2023).

[14] Ghosh S., Kim S., Ijaz M.F., Singh P.K., Mahmud M. (2022). Classification of mental stress from wearable physiological sensors using image-encoding-based deep neural network. Biosensors.

[15] Mukherjee S.K., Dave M., Singh P.K. (2024). A Transfer Learning Approach for the Classification of Human Emotions Using Electroencephalogram Signals. International Conference on Emerging Applications of Information Technology.

[16] Anjum M.F., Dasgupta S., Mudumbai R., Singh A., Cavanagh J.F., Narayanan N.S. (2020). Linear predictive coding distinguishes spectral EEG features of Parkinson’s disease. Park. Relat. Disord..

[17] Lee S.-B., Kim Y.-J., Hwang S., Son H., Lee S.K., Park K.-I., Kim Y.-G. (2022). Predicting Parkinson’s disease using gradient boosting decision tree models with electroencephalography signals. Park. Relat. Disord..

[18] Lal U., Chikkankod A.V., Longo L. (2024). Fractal dimensions and machine learning for detection of Parkinson’s disease in resting-state electroencephalography. Neural Comput. Applic..

[19] Latifoğlu F., Penekli S., Orhanbulucu F., Chowdhury M.E.H. (2024). A novel approach for Parkinson’s disease detection using Vold-Kalman order filtering and machine learning algorithms. Neural Comput. Appl..

[20] Loh H.W., Ooi C.P., Palmer E., Barua P.D., Dogan S., Tuncer T., Baygin M., Acharya U.R. (2021). GaborPDNet: Gabor transformation and deep neural network for parkinson’s disease detection using EEG signals. Electronics.

[21] Qiu L., Li J., Pan J. (2022). Parkinson’s disease detection based on multi-pattern analysis and multi-scale convolutional neural networks. Front. Neurosci..

[22] Qiu L., Li J., Zhong L., Feng W., Zhou C., Pan J. (2024). A novel EEG-Based Parkinson’s disease detection model using multiscale convolutional prototype networks. IEEE Trans. Instrum. Meas..

[23] Chang H., Liu B., Zong Y., Lu C., Wang X. (2023). EEG-Based Parkinson’s disease recognition via Attention-Based Sparse Graph Convolutional Neural Network. IEEE J. Biomed. Health Inform..

[24] OpenNeuro. https://openneuro.org/datasets/ds002778/versions/1.0.5.

[25] Gramfort A. (2013). MEG and EEG data analysis with MNE-Python. Front. Neurosci..

[26] Ksibi A., Zakariah M., Menzli L.J., Saidani O., Almuqren L., Hanafieh R.A.M. (2023). Electroencephalography-Based depression detection using multiple machine learning techniques. Diagnostics.

[27] Mukherjee D., Mondal R., Singh P.K., Sarkar R., Bhattacharjee D. (2020). EnsemConvNet: A deep learning approach for human activity recognition using smartphone sensors for healthcare applications. Multimed. Tools Appl..

[28] Bhattacharya D., Sharma D., Kim W., Ijaz M.F., Singh P.K. (2022). Ensem-HAR: An Ensemble Deep Learning Model for Smartphone Sensor-Based Human Activity Recognition for Measurement of Elderly Health Monitoring. Biosensors.

[29] Banerjee A., Sarkar A., Roy S., Singh P.K., Sarkar R. (2022). COVID-19 chest X-ray detection through blending ensemble of CNN snapshots. Biomed. Signal Process. Control.

[30] Tanveer M., Rashid A.H., Kumar R., Balasubramanian R. (2022). Parkinson’s disease diagnosis using neural networks: Survey and comprehensive evaluation. Inf. Process. Manag..

[31] Goutte C., Gaussier E. (2005). A Probabilistic Interpretation of Precision, Recall and F-Score, with Implication for Evaluation. European Conference on Information Retrieval.

[32] Lee S., Liu A., Wang Z.J., McKeown M.J. (2019). Abnormal phase coupling in parkinson’s disease and normalization effects of subthreshold vestibular stimulation. Front. Hum. Neurosci..

